# 77822 PSD95-nNOS interaction alters the basolateral amygdala transcriptome following fear conditioning: implications for molecular mechanisms underlying PTSD

**DOI:** 10.1017/cts.2021.463

**Published:** 2021-03-30

**Authors:** Jheel Patel, Melissa Haulcomb, Liangping Li, Guanglong Jiang, Erik Dustrude, Yunlong Liu, Yvonne Lai, Andrei Molosh, Anantha Shekhar

**Affiliations:** Indiana University School of Medicine

## Abstract

ABSTRACT IMPACT: This research takes a transcriptomic approach to parse genes and molecular pathways that underlie the fear memory circuitry and, in doing so, identifies therapeutic targets that can further be developed into treatments for fear disorders, such as post-traumatic stress disorder. OBJECTIVES/GOALS: Normal fear learning produces avoidance behavior that promotes survival, but excessive and persistent fear after trauma can lead to development of phobias and post-traumatic stress disorder (PTSD). Our goal is to understand the mechanism and identify novel genetic targets underlying fear responses. METHODS/STUDY POPULATION: Involvement of the basolateral amygdala (BLA) in fear acquisition is well established and requires activation of N-methyl-D-aspartic acid receptors (NMDARs). At a cellular level, NMDAR activation leads to production of nitric oxide (NO) by a process mediated by interaction between postsynaptic density protein 95 (PSD95) and neuronal nitric oxide synthase (nNOS). To elucidate mechanisms underlying the role of the PSD95-nNOS-NO pathway in conditioned fear, here we use rodent behavioral paradigms, pharmacological treatment with a small molecule PSD95-nNOS inhibitor, RNA-sequencing, and an AAV-mediated knockdown of the nNOS gene in the BLA. RESULTS/ANTICIPATED RESULTS: We show that treatment with ZL006 attenuates rodent cued-fear consolidation. Additionally, with RNA-sequencing, expression of 516 genes was altered in the BLA following fear expression; of these genes, 83 were restored with systemic ZL006 treatment. Network data and gene ontology enrichment analyses further revealed that cGMP effects, insulin-like growth factor binding, and cognition-related pathways were significantly altered. Finally, we show that a BLA-specific knockdown of nNOS attenuates cued fear consolidation, without adverse effects on other memory and motor behaviors. DISCUSSION/SIGNIFICANCE OF FINDINGS: Via a model of NMDAR-mediated fear consolidation, our results reveal novel pathways and genetic targets that underlie plasticity of fear memory circuitry. Importantly, these results will inform future therapeutic strategies for targeting fear related disorders like PTSD.

